# The correlation of p22^phox^ and chemosensitivity in EGFR-TKI resistant lung adenocarcinoma

**DOI:** 10.18632/oncotarget.26637

**Published:** 2019-02-01

**Authors:** Masayuki Kobayashi, Ryoko Saito, Yasuhiro Miki, Ren Nanamiya, Chihiro Inoue, Jiro Abe, Ikuro Sato, Yoshinori Okada, Hironobu Sasano

**Affiliations:** ^1^ Department of Pathology, Tohoku University Graduate School of Medicine, Miyagi, Japan; ^2^ Department of Thoracic Surgery, Miyagi Cancer Center, Miyagi, Japan; ^3^ Department of Pathology, Miyagi Cancer Center, Miyagi, Japan; ^4^ Department of Thoracic Surgery, Tohoku University Hospital, Miyagi, Japan

**Keywords:** EGFR-TKI resistance, chemoresistance, p22^phox^, HIF-1α, EMT

## Abstract

**Background:**

Enhancing the chemosensitivity in the patients with epidermal growth factor receptor-tyrosine kinase inhibitor (EGFR-TKI) resistant lung adenocarcinoma (LUAD) is pivotal in achieving their successful therapeutic outcome. We aimed to explore the mechanisms regarding the development of therapeutic resistance to chemotherapy in EGFR-TKI resistant LUAD. Methods: Microarray analysis lead to potential involvement of p22^phox^, which was abundantly expressed in the cell lines harboring EGFR-TKI resistance and chemoresistance, and was known to regulate several important chemoresistance-associated factors such as hypoxia inducible factor-1α (HIF-1α) and epithelial-mesenchymal transition (EMT). We compared the status of p22^phox^ with that of chemoresistance, HIF-1α expression and EMT in LUAD cell lines. We immunolocalized p22^phox^ in the specimens of lung cancer patients.

**Results:**

p22^phox^ and HIF-1α mRNAs were significantly elevated in the cells harboring EMT and chemoresistance. p22^phox^ knockdown enhanced chemosensitivity and reduced the expression of HIF-1α and EMT-associated factors. HIF-1α knockdown enhanced the chemosensitivity, while HIF-1α transfection induced EMT and chemoresistance in these cell lines. All LUAD patients with T790M mutation were associated with abundant p22^phox^ immunoreactivity in carcinoma cells.

**Conclusions:**

The analysis of p22^phox^ in lung carcinoma tissues could provide new insights into the selection of chemotherapy for the patients with EGFR-TKI resistant LUAD.

## INTRODUCTION

Lung cancer is one of the most lethal cancers worldwide. Epidermal growth factor receptor (EGFR) gene mutation is known as one of the driver mutations of lung adenocarcinoma (LUAD) and LUAD patients harboring EGFR mutation account for more than half of LUAD patients in Asia [1]. EGFR–tyrosine kinase inhibitors (EGFR–TKIs), such as gefitinib and erlotinib, have been demonstrated to be effective for the patients with EGFR mutation [2, 3]. However, acquired resistance to EGFR-TKIs is practically unavoidable and is generally considered to occur as a result of gatekeeper mutations including a threonine-to-methionine substitution on codon 790 (T790M) [4]. It is true that effective EGFR-TKI was recently developed for the patients with LUAD harboring T790M mutation [5], but other mechanisms of therapeutic resistance to this EGFR-TKI through acquiring further mutations have been also reported [6]. Cytotoxic anti-cancer drugs have been widely used for those with LUAD acquiring EGFR-TKI resistance. However, to the best of our knowledge, the possible influence of EGFR-TKI resistance on development of chemoresistance in LUAD has remained virtually unknown because the relationship between EGFR-TKI resistance and chemotherapy resistance has not necessarily been studied in LUAD [7]. Therefore, in this study, we first aimed to study this potential correlation above in LUAD.

It is also true that numerous factors associated with chemoresistance have been reported in the literature. For instance, hypoxia inducible factor-1α (HIF-1α) and epithelial-mesenchymal transition (EMT) were both reported to induce chemoresistance in various human malignancies [7, 8]. HIF-1α is a well-known transcription factor activated in hypoxia state. However, its activation was reported as a result of an activation of the phosphoinositide 3-kinase (PI3K) / Akt pathway [9] under normoxia and numerous examples of HIF-1α activation without hypoxia have been since reported. For instance, HIF-1α was demonstrated to induce chemotherapy resistance in lung cancer under both hypoxia and normoxia state [10]. In addition, an involvement of multiple drug resistance 1, BCL2 / adenovirus E1B 19 kDa protein-interacting protein 3 and EMT have been reported to be associated with HIF-1α-induced chemoresistance [11–13]. In particular, an induction of EMT was reported to decrease the therapeutic efficacy of the first generation EGFR-TKI [7] and cytotoxic anti-cancer drugs in lung cancer patients [8]. However, the possible involvement of HIF-1α and EMT in EGFR-TKI resistance in chemotherapy resistance has remained virtually unknown.

p22^phox^ forms a heterodimer with nicotinamide adenine dinucleotide phosphate (NADPH) oxidase (NOX), one of the main reactive oxygen species (ROS) generators, in order to stabilize the cell membrane [14]. p22^phox^ was also recently reported to increase cell survival and proliferation in various human malignancies. For instance, in pancreatic cancer, upregulation of p22^phox^ was reported to inhibit apoptosis by ROS [15]. In prostate cancer, upregulation of p22^phox^ was also reported to activate PI3K/Akt pathway and extracellular signal-regulated kinase 1/2 (ERK1/2) pathway and subsequently induce HIF-1α via ROS signaling [16]. In oral squamous cell carcinoma, p22^phox^ overexpression was reported to inhibit cisplatin (CDDP)-induced apoptosis. Results of these studies above all indicated that p22^phox^ status was indeed correlated with chemoresistance in several human malignancies. In non-small cell lung cancer (NSCLC), an increased expression of p22^phox^ was reported to cause EMT [17]. However, the possible roles of p22^phox^ in lung cancer after acquired resistance to EGFR-TKI have not been known. In addition, overexpression of NOX2 was reported to induce ROS in NSCLC with EGFR-TKI resistance [18]. Therefore, in this study, we firstly studied whether p22^phox^ could possibly contribute to the development of chemoresistance via HIF-1α and EMT in LUAD harboring acquired resistance to EGFR-TKI or not and elucidated the possible development of new treatments to overcome the therapeutic resistance in these patients.

## RESULTS

### p22^phox^ was upregulated in chemoresistant LUAD after acquired resistance to EGFR-TKI

**Figure 1:**
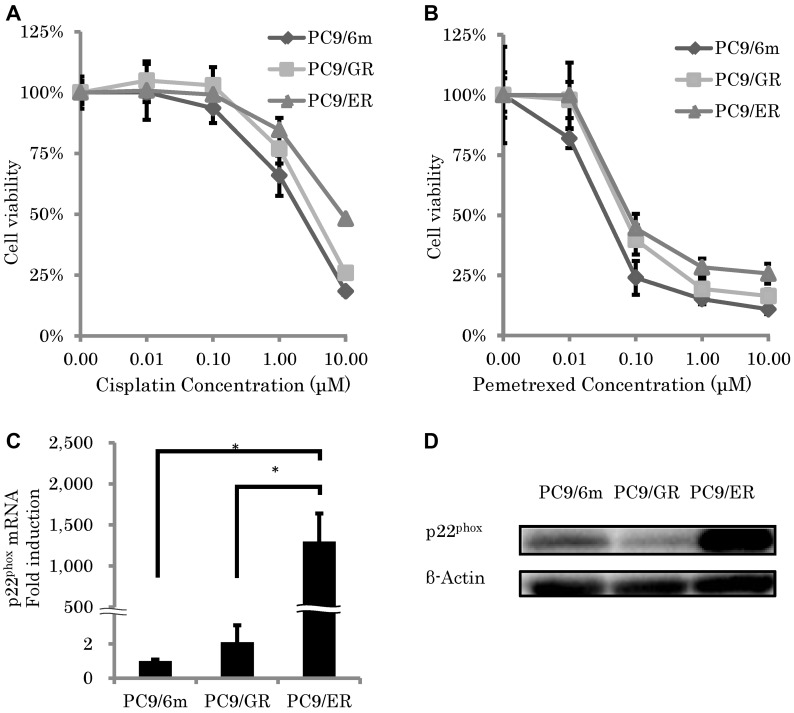
Chemosensitivity and expression of p22^phox^ in epidermal growth factor receptor–tyrosine kinase inhibitor (EGFR-TKI)–resistant lung adenocarcinoma cells (**A**, **B**) Cell viability was measured using WST-8 assay of control lung adenocarcinoma cell line (PC9/6m) and EGFR-TKI resistant lung adenocarcinoma cell lines (PC9/GR and PC9/ER) treated with cisplatin (A) and pemetrexed (B) for 72 h; *N* = 4. (**C**, **D**) Expression level of p22^phox^. mRNA levels (*N* = 3) (C) and protein expressions (D) of p22^phox^ in high chemoresistant cell line (PC9/ER) were significantly higher than chemo-sensitive cell line (PC9/6m) and low chemoresistance cell line (PC9/GR). The significance of difference between indicated groups are calculated by Student's *t*-test (^*^P < 0.05).

We first performed cell viability assays after CDDP or pemetrexed (PEM) treatment using the WST-8 Cell Counting Kit-8. Results of cell viability in CDDP (1 μM and 10 μM) and PEM (0.01 μM, 0.1 μM, 1μM and 10 μM) treatment for 3 days in both PC9/GR and PC9/ER were significantly higher than those in PC9/6m (PC9/GR: *P* = 0.0421, 0.0003, 0.0091, 0.0007, 0.0491, 0.0070, PC9/ER: *P* = 0.003, *P* < 0.0001, *P* = 0.0044, *P* < 0.0001, *P* < 0.0001, *P* < 0.0001, respectively) (Figure 1A and 1B, respectively). In addition, in the cells treated with CDDP (10 μM) and PEM (1 μM and 10μM), the cell viability in PC9/ER was significantly higher than that in PC9/GR (*P* = 0.003, 0.0004, *P* < 0.0001, respectively) (Figure 1A and 1B). Therefore, those results above indicated that PC9/ER was highly chemoresistant LUAD cell line following acquired resistance to EGFR-TKI. Therefore, we performed comprehensive gene analysis by microarray in order to further study gene profiling of PC9/ER. Among the factors associated with HIF-1α pathway or EMT induction, both of which are well known to induce chemoresistance in several human malignancies, the status of p22^phox^ in PC9/ER was particularly higher than that in PC9/6m and PC9/GR. The amounts of p22^phox^ expression at both mRNA and protein levels were significantly higher in PC9/ER than those in both PC9/6m and PC9/GR (mRNA; *P* = 0.0002, 0.0002, respectively) (Figure 1C and 1D).

### Knockdown of p22^phox^ enhanced efficiency of chemotherapy in EGFR-TKI resistant LUAD cell lines harboring EGFR T790M mutation

**Figure 2:**
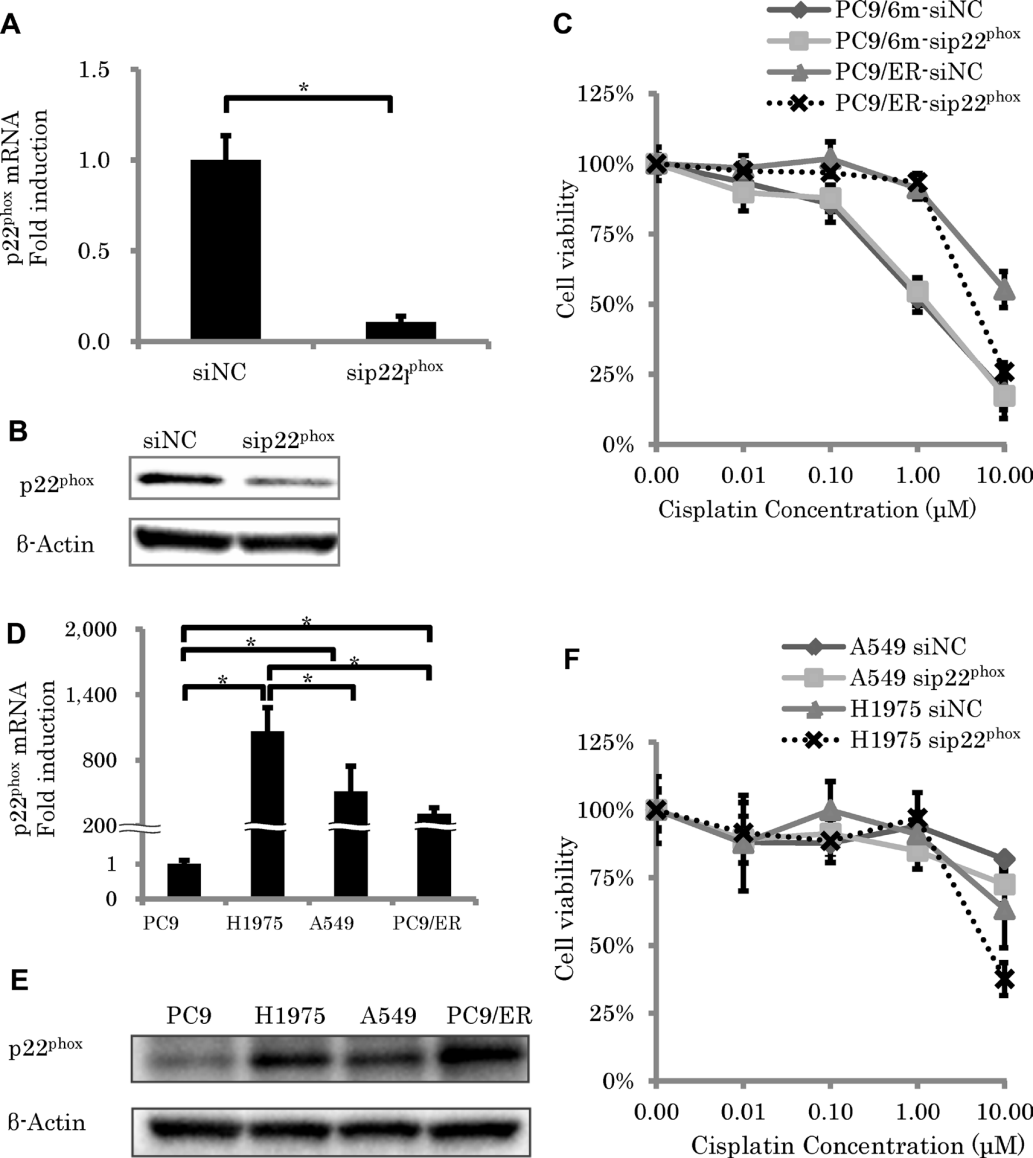
The effect of p22^phox^ knockdown on sensitivity to cisplatin-induced cytotoxicity (**A**, **B**) Expression level of p22^phox^ mRNA (*N* = 3) (A) and protein expressions of p22^phox^ (B) in EGFR-TKI and chemotherapy resistant lung adenocarcinoma cells (PC9/ER) were significantly knocked down by siRNA (5 nM). (**C**) Cell viability was measured using WST-8 assay in control lung adenocarcinoma cells (PC9/6m) and PC9/ER transfected with siRNA (5 nM) for 24h, followed by treatment with cisplatin for another 48 h (*N* = 4). (**D**, **E**) Expression level of p22^phox^. mRNA levels (*N* = 3) (D) and protein expressions (E) of p22^phox^ in EGFR-TKI resistant cells (H1975) was significantly higher than PC9, A549 and PC9/ER. (**F**) Cell viability was measured using WST-8 assay in H1975 and A549 transfected with siRNA (5 nM) for 24h, followed by treatment with cisplatin for another 48 h (*N* = 4). The significance of difference between indicated groups are calculated by Student's *t*-test (^*^P < 0.05).

To examine the roles of p22^phox^ against chemoresistance in EGFR-TKI resistant LUAD, we performed knockdown of p22^phox^ expression by using siRNA (mRNA; *P* = 0.0004) (Figure 2A and 2B). Results of the cell viability assay did demonstrate that knockdown of p22^phox^ significantly enhanced efficiency of CDDP cytotoxicity (10 μM) in PC9/ER (*P* < 0.0001), but not in PC9/6m (*P* = 0.1704) (Figure 2C). Therefore, in order to further confirm whether the effects of p22^phox^ on chemosensitivity was inherent to PC9/ER or not, we evaluated the effects of p22^phox^ on other EGFR-TKI resistant LUAD cell lines. The amounts of p22^phox^ expression at both mRNA and protein levels in H1975 and A549 were significantly higher than those in PC9 (mRNA; *P* < 0.0001, *P* = 0.0045, respectively) (Figure 2D and 2E). Knockdown of p22^phox^ significantly enhanced efficiency of CDDP cytotoxicity (10 μM) in H1975 (*P* = 0.0202) but not in A549 (*P* = 0.0556) (Figure 2F).

### p22^phox^ regulated HIF-1α in EGFR-TKI resistant LUAD cell lines

**Figure 3:**
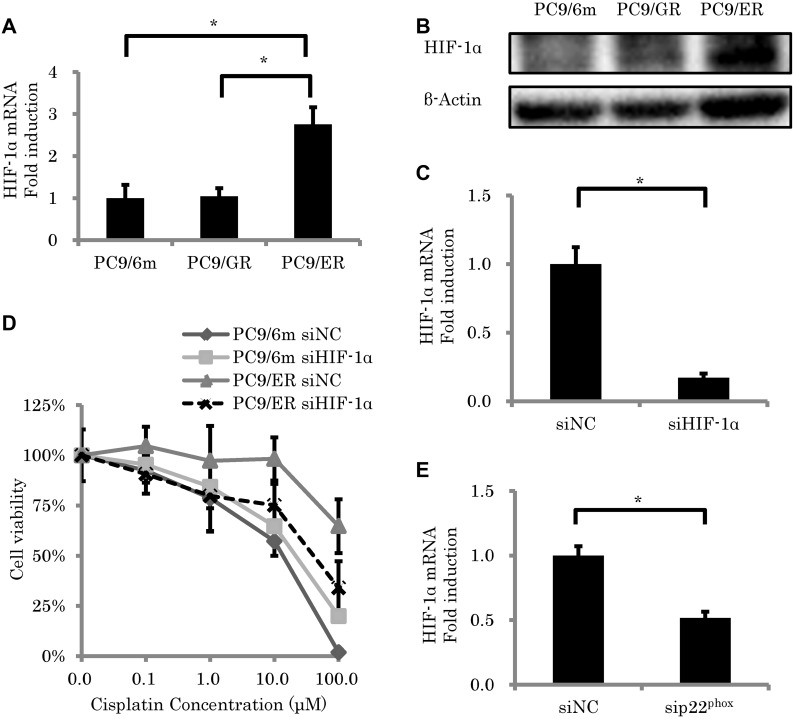
The effect of HIF-1α knockdown on sensitivity to cisplatin-induced cytotoxicity (**A**, **B**) Expression level of HIF-1α. mRNA levels (*N* = 3) (A) and protein expressions (B) of HIF-1α in high chemoresistant cell line (PC9/ER) were significantly higher than in chemo-sensitive cells (PC9/6m) and in low chemoresistant cells (PC9/GR). (**C**) The expression of HIF-1α in PC9/ER was knocked down by siRNA (5 nM) (*N* = 3). (**D**) Cell viability was measured using WST-8 assay in control lung adenocarcinoma cell line (PC9/6m) and PC9/ER transfected with siRNA (5 nM) for 24h, followed by treatment with cisplatin for another 48 h (*N* = 4). (**E**) HIF-1α expression was significantly decreased by knockdown of p22^phox^ in PC9/ER. The significance of difference between indicated groups are calculated by Student's *t*-test (^*^P < 0.05).

We then focused on HIF-1α, because this protein was reported to contribute enormously to the development of chemoresistance [19] through an induction by p22^phox^ via ROS [16], in order to further clarify the downstream of p22^phox^ in the effect of chemoresistance. The amounts of HIF-1α expression at both mRNA and protein levels in PC9/ER were significantly higher than those in both PC9/6m and PC9/GR (mRNA; *P* = 0.0005, 0.0006, respectively) (Figure 3A and 3B, Supplementary Figure 1). We therefore performed knockdown of HIF-1α expression (*P* = 0.0003) (Figure 3C) in order to further examine the effects of HIF-1α on the development of chemoresistance in EGFR-TKI resistant LUAD. Results of the cell viability assay did demonstrate that the knockdown of HIF-1α significantly decreased the survival rate in PC9/ER (0.1 μM, 1 μM, 10 μM and 100 μM) (*P* = 0.0270, 0.0423, 0.0006, 0.0003, respectively) (Figure 3D). In addition, in order to clarify whether p22^phox^ induced the expression of HIF-1α or not, we subsequently performed the knockdown of p22^phox^ using its corresponding siRNA. Results did demonstrate that the knockdown of p22^phox^ decreased the HIF-1α mRNA expression in PC9/ER (*P* = 0.0007) (Figure 3E).

### p22^phox^ induced EMT in EGFR-TKI resistant LUAD cell lines

**Figure 4:**
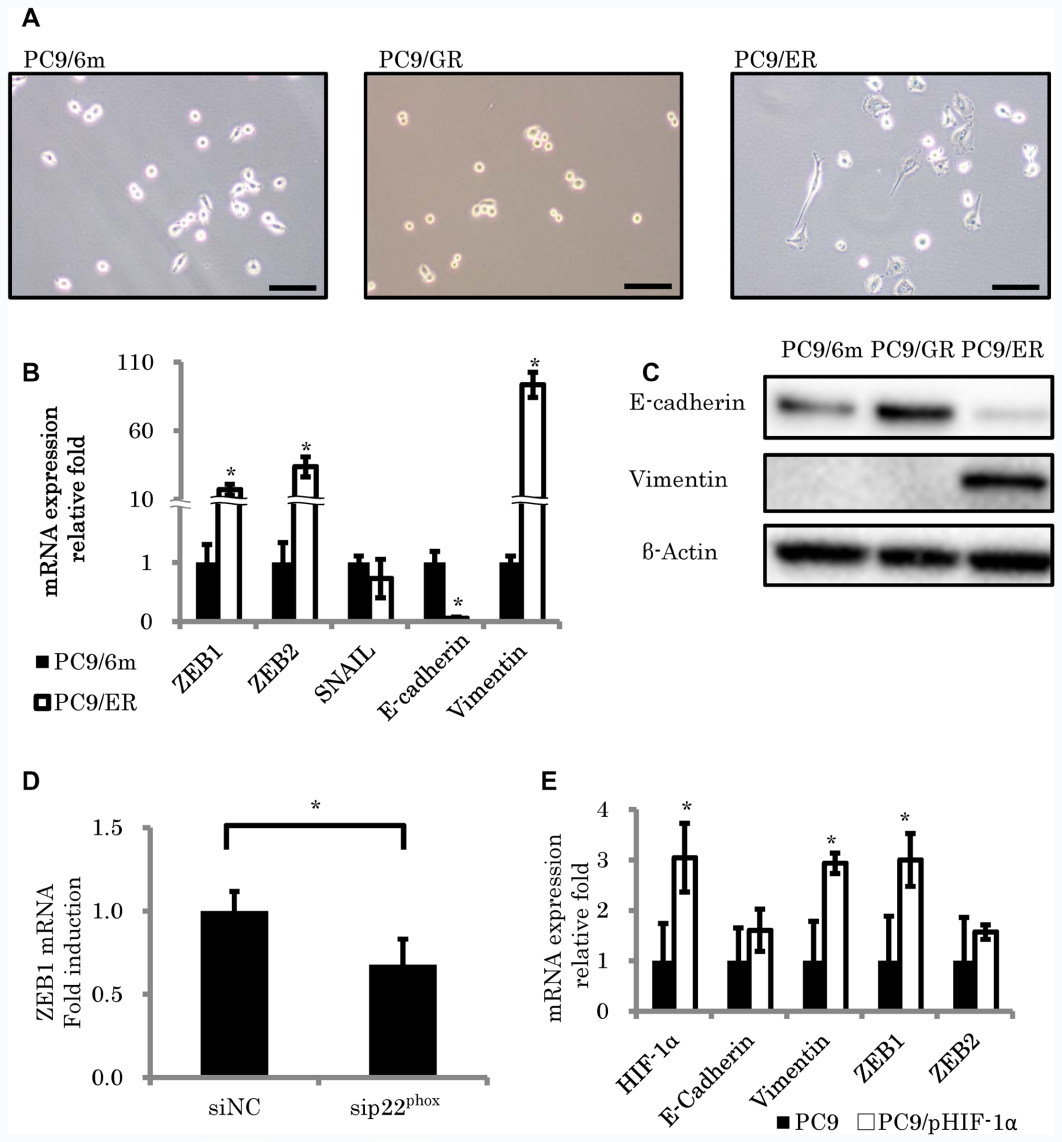
Morphologic changes consistent with epithelial to mesenchymal transition (EMT) in EGFR-TKI resistant lung adenocarcinoma cell lines (**A**) Morphological features of control cells (PC9/6m) and EGFR-TKI resistant cell lines (PC9/GR, PC9/ER). PC9/ER demonstrated spindle cell appearance, which was consistent with EMT. (**B**, **C**) Expression of EMT-related factors. mRNA levels (*N* = 3) (B) and protein expressions (C) in EGFR-TKI resistant cells. (**D**) ZEB1 expression was significantly decreased by knockdown of p22^phox^ in PC9/ER. (**E**) The effect of HIF-1α overexpression on EMT-related gene expressions in PC9. Both vimentin and ZEB1 levels were significantly increased in stable clones with high HIF-1α expression (PC9/pHIF-1α). The significance of difference between indicated groups are calculated by Student's *t*-test (^*^P < 0.05).

The cells demonstrating spindle cell morphology were detected only in PC9/ER (Figure 4A). In addition, the expression of epithelial biomarker (E-cadherin) was significantly decreased (mRNA; *P* = 0.001) and the levels of mesenchymal proteins (Vimentin) and EMT inducible proteins, zinc finger E-box-binding homeobox 1 (ZEB1) and ZEB2, were also significantly increased in PC9/ER than PC9/6m (mRNA; *P* < 0.001, *P* = 0.0023, 0.0014, respectively) (Figure 4B and 4C). These results all indicated that EMT was indeed induced in PC9/ER. EMT was reported to contribute to the development of chemoresistance [8], we then studied the effects of p22^phox^ and/or HIF-1α on EMT. Results demonstrated that the knockdown of p22^phox^ by employing its corresponding siRNA significantly decreased the mRNA expression of ZEB1 (*P* = 0.0448) (Figure 4D). In addition, in order to further clarify the association of HIF-1α with EMT, we transfected plasmid of HIF-1α into PC9 and examined the expression of EMT-related genes. Both vimentin and ZEB1 levels were significantly increased by HIF-1α overexpression (*P* = 0.0144, 0.0282, respectively) (Figure 4E).

### Upregulation of p22^phox^ expression in EGFR-TKI resistant LUAD tissue

**Figure 5:**
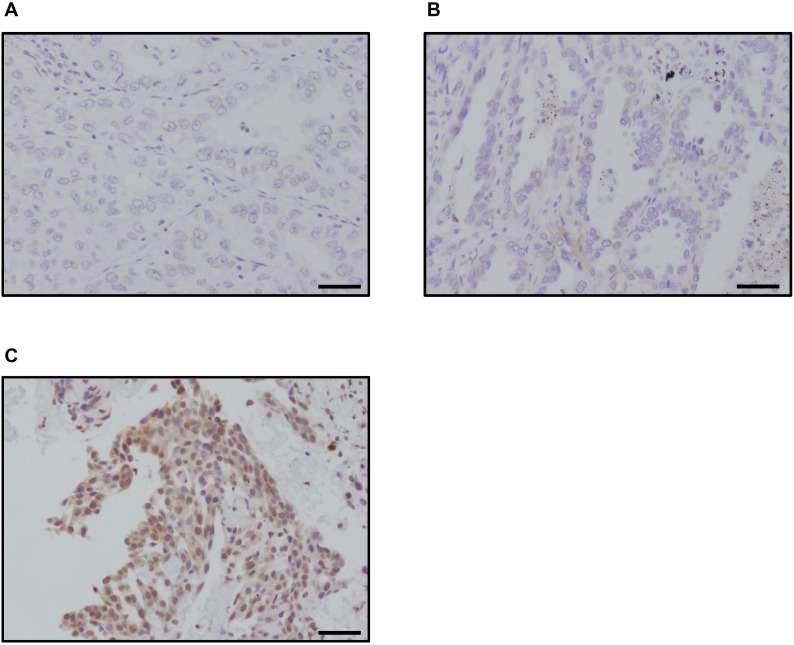
Immunohistochemistry of p22^phox^ in patients with lung adenocarcinoma (**A**–**C**) Representative findings of p22^phox^ immunohistochemistry in patients with lung adenocarcinoma (bar: 50 μm). p22^phox^ immunoreactivity was detected in the cytoplasm of carcinoma cells: (A) negative (T790M mutation negative), (B) weakly stained (T790M mutation negative), (C) strongly stained (T790M mutation positive).

We immunolocalized p22^phox^ in clinical specimens of lung cancer in order to clarify the possible association of p22^phox^ with clinicopathological factors of LUAD patients. Representative findings were presented in Figure 5A–5C. The great majority of T790M mutation negative LUAD were immunohistochemically negative for p22^phox^ (Figure 5A) and only a few p22^phox^ weakly positive carcinoma cells (Figure 5B) were detected in nine of 53 cases (Table 1A). No significant correlations between p22^phox^ expression and any of the clinicopathological factors examined in T790M mutation negative 53 cases. A platinum based adjuvant chemotherapy was performed for nine patients, however, the tumor were resected completely in all nine cases. Therefore, we could not examine the association between p22^phox^ expression and chemosensitivity. We additionally examined six cases which harbored T790M mutated EGFR. All of these T790M mutated LUAD cases were markedly immunopositive for p22^phox^ (Figure 5C, Table 1B). These results did indicate the significant association between p22^phox^ and T790M mutation in LUAD. In addition, two cases harboring primary T790M mutated EGFR did demonstrate rather weak E-cadherin immunoreactivity, although these two cases did not necessarily demonstrate the histological features of spindle cells and vimentin immunoreactivity. All four cases harboring acquired T790M mutated EGFR were immunohistochemically positive for vimentin. In addition, three of these four cases had foci of spindle-shaped tumor cells, and two of them were weakly positive for E-cadherin (Supplementary Figure 2). These six cases harboring EGFR-TKI resistance due to T790M mutated EGFR and p22^phox^ immunoreactivities were therefore considered at the stage of progressing to EMT.

Table 1(A) Association between p22^phox^ immunoreactivity and clinicopathological factors in patients with lung adenocarcinoma (*N* = 53)

**Table:** 

	p22^phox^ immunoreactivity		
	negative (*N* = 44)	low (*N* = 9)	*P* value
Age	68.2 ± 1.4	69.6 ± 2.5	0.692
Sex			0.8662
Male	22	7	
Female	22	2	
Brinkman Index	367.0 ± 477.7	298.8 ± 343.8	0.7025
pStage			0.6091
I	30	8	
II	6	0	
III	8	1	
pT			0.3854
1	29	4	
2	14	5	
3	1	0	
pN			0.6652
0	33	8	
1–3	11	1	
EGFR mutation			0.456
positive	16	5	
negative	28	4	

Data are presented as average ± standard deviation, ^*^P value < 0.05 significant. pT; pathological T factor; pN: pathological N factor; pStage: pathological Stage.

**Table:** (B) Association between p22^phox^ immunoreactivity and EGFR T790M mutation in lung adenocarcinoma patients harboring EGFR mutation (*N* =27)

	p22^phox^ immunoreactivity			
	negative (*N* = 16)	low (*N* = 5)	high (*N* = 6)	*P* value
T790M mutation				< 0.001
positive (*N* = 6)	0	0	6	
negative (*N* = 21)	16	5	0	

Data are presented as 0% (negative); 1–50% (low); 50% < (high), ^*^P value < 0.05 significant.

## DISCUSSION

To the best of our knowledge, this is the first study to evaluate the mechanisms of chemotherapy resistance induced as a result of acquired resistance to EGFR-TKI. Results of our present study did clearly demonstrate that p22^phox^ influenced the status of chemosensitivity in EGFR-TKI resistant LUAD cell. In addition, p22^phox^ abundant LUAD cells did acquire the features of chemoresistance via pathways such as HIF-1α and EMT. This study is the first report to fully investigate the significance of p22^phox^ expression and its functions in LUAD acquiring EGFR-TKI resistance following EGFR-TKI therapy.

Results of comprehensive gene expression analysis by microarray also did demonstrate that p22^phox^ was markedly increased in EGFR-TKI-resistant LUAD cell lines, which acquired therapeutic resistance to both EGFR-TKIs and cytotoxic anticancer drugs by long-term exposure of first generation EGFR-TKI (gefitinib and erlotinib). In addition, results of cell viability assays also demonstrated that p22^phox^ knockdown by its corresponding siRNA increased therapeutic sensitivity to CDDP therapy in p22^phox^ high LUAD cell lines. Therefore, these results all indicated that the increased expression of p22^phox^ did contribute to the acquisition or development of chemoresistance in EGFR-TKI-resistant LUAD cells. In addition, the findings that the increased HIF-1α expression and the presence of EMT detected in p22^phox^ abundant LUAD did demonstrate that p22^phox^ could regulate HIF-1α pathway and EMT induction in EGFR-TKI-resistant LUAD, subsequently resulting in the process of acquisition of chemoresistance. These results were also consistent with the results of previous reports that increased p22^phox^ contributed to carcinoma cell survival in some human malignancies. For instance, upregulation of p22^phox^ was reported to activate both PI3K/Akt and ERK1/2 pathways and induce HIF-1α via ROS signaling in prostate cancer [16]; overexpression of p22^phox^ was also reported to cause diminishing the entry of CDDP into the nuclei followed by an inhibition of apoptosis and drug resistance in oral squamous cell carcinoma [20]; EMT was also reported to be induced when p22^phox^ expression was increased by exposure to tobacco smoke extract in NSCLC [17]. However, further comprehensive study to explore the possible correlation between p22^phox^ and EMT is required for further clarification.

The A549 cell line exhibited high chemotherapy resistance and increased expression of p22^phox^ mRNA in the same fashion as EGFR-TKI-resistant LUAD cell lines but p22^phox^ knockdown did not attenuate chemoresistance in A549, in contrast to EGFR-TKI-resistant LUAD cell lines. This could be due to the difference of oncogene status. In detail, both EGFR-TKI-resistant LUAD cell lines, PC9/ER and H1975, had the common EGFR gene mutation (T790M and L858R mutation) [21], but A549 had wild-type EGFR. Therefore, some different mechanisms of chemoresistance in addition to p22^phox^ pathway or EGFR gene mutation were reasonably postulated to occur in A549 but it awaits further investigations for clarification.

It was reported that when T790M mutation occurred in EGFR, it formed a complex with NOX [18, 22], which could then contribute to increased its expression and stability of p22^phox^ protein but the association between EGFR pathway or minor mutations and p22^phox^ may be required for confirmation.

We also immunolocalized p22^phox^ in LUAD without EGFR-TKI treatment and T790M mutation. The detailed status of p22^phox^ has not been studied in human LUAD. However, p22^phox^ was reported to be more abundant in non-small cell lung carcinoma cells following the exposure to tobacco smoke extract [17], stress signal inducers such as smoking were considered to influence its expression. However further investigations are required to clarify the mechanisms of p22^phox^ induction in lung cancer. In our present study, p22^phox^ was expressed in only a few LUAD patients before acquisition of EGFR-TKI resistance and T790M mutation. In addition, even if p22^phox^ was positive in these cases, the percentage of p22^phox^ positive cells in the tumor cells was extremely low and the intensity was really weak. Therefore, the expressions of p22^phox^ in naive LUAD tumor cells are relatively low. In addition, there were no significant differences of p22^phox^ immunoreactivity between EGFR mutation positive and negative cases examined. As discussed above, other factors were considered to be associated with p22^phox^ expression in naive LUAD. However, EGFR-TKI resistance or T790M mutation in carcinoma cells itself could induce p22^phox^ and the status of p22^phox^ in LUAD tissues without T790M mutation was by no means correlated with any of the clinicopathological factors examined. On the other hand, p22^phox^ was significantly elevated in LUAD patients harboring T790M mutation, and these six cases demonstrated partial EMT status. Results of both *in vivo* and *in vitro* studies did indicate that p22^phox^ expression could be correlated with development of chemoresistance and EMT in EGFR-TKI resistant LUAD possibly through T790M mutation. However, to our regret, the clinical specimens associated with EGFR-TKI resistant LUAD were not necessarily available in sufficient quantity in our present study. Therefore the correlation between T790M mutation and the potential roles of p22^phox^ should be clarified by further studies.

In summary, this is the first study to demonstrate how chemoresistance could be induced in EGFR-TKI resistant LUAD in detail. Results did demonstrate that p22^phox^ was an important factor influencing chemoresistance in EGFR-TKI resistant LUAD. p22^phox^ also contributed to the development of chemoresistance partially via HIF-1α and EMT. These results will provide new insights into the development of therapeutic strategy for the patients with EGFR-TKI–resistant LUAD, for example, treatment targeting p22^phox^ could possibly attenuate the chemotherapy resistance of LUAD which acquired resistance to EGFR-TKI.

## MATERIALS AND METHODS

### Reagents and antibodies

The following materials were commercially obtained: Gefitinib (Biaffin, Kassel, Germany); Erlotinib (kindly provided from Roche Diagnostics, Mannheim, Germany); Cisplatin (Wako Pure Chemical Industries, Osaka, Japan); Pemetrexed (Wako Pure Chemical Industries); TGF-β1 (PeproTech, Rocky Hill, USA).

Antibodies were obtained from the following sources: p22^phox^ [E-11] (Santa Cruz Biotechnology, Santa Cruz, CA, USA); HIF-1α [h1alpha67] (Abcam, Cambridge, UK), E-cadherin [4A2C7] (Thermo Fisher Scientific, Rockford, USA), Vimentin [V9] (Acris, San Diego, USA), β-actin [AC-15] (Sigma-Aldrich, St. Louis, MO, USA).

### Cell culture

Human LUAD cell lines used in this study are as follows: PC9 (Riken Cell Bank, Tsukuba, Japan), NCI-H1975 (H1975) and A549 (American Type Cell Culture Collection, Manassas, VA, USA) and PC9/6m, PC9/ER, and PC9/GR were established in our laboratory previously [23]. PC9 (Immuno-biological Laboratories, Gunma, Japan) was exposed to increasing concentrations of gefitinib and erlotinib to generate the gefitinib- and erlotinib-resistant cell lines (PC9/GR and PC9/ER, respectively). These doses were gradually increased to 10 nM (2 months), 1 μM (2 months), and 5 μM (2 months). PC9 cells were also cultured for 6 months (PC9/6m) in regular medium to eliminate the effects of long-term cell culture. These cells have EGFR mutation as follows: PC9, PC9/6m, and PC9/GR: exon 19 deletion; PC9/ER: exon 19 deletion, L858R mutation, and T790M mutation.

These cells were maintained in Roswell Park Memorial Institute media (RPMI) 1640 (Sigma-Aldrich) supplemented with 10% fetal bovine serum (FBS) (Nichirei, Tokyo, Japan) and 1% penicillin/streptomycin at 37° C in a humidified incubator containing 5% CO_2_.

### Microarray

Total RNA was extracted with care from PC9/6m, PC9/GR, and PC9/ER as described below. Next, 44 K Whole Human genome arrays (G4112F) (Agilent Technologies, Santa Clara, CA, USA) were prepared and hybridized with linearly amplified and labeled total RNA, according to the manufacturer's protocol. Complementary RNA (cRNA) probes were labeled using a Low Input Linear Amplification and Labeling Kit (Agilent Technologies). Fluorescently labeled probes were purified using an RNeasy Mini Kit (Qiagen, Hilden, Germany), according to the manufacturer's instructions. Results were extracted using Feature Extraction software (Agilent Technologies) and were analyzed using Genespring GX11 software (Agilent Technologies) to obtain gene expression ratios.

### Cell viability assays

Two and/or three-day cell viability assays were carried out by plating 3000 cells per well of each cell lines into 96-well plates. The following day, each cell was treated with each drug across a 4-dose range from 1 nM to 10 μM. After 48h or 72 h of drug treatment, cell viability was measured using WST-8 Cell Counting Kit-8 (Dojindo Laboratories, Kumamoto, Japan).

### Real-Time RT-PCR

Total RNA was extracted carefully from cultured cells using TRI reagent (Molecular Research Center, Cincinnati, OH, USA) and was reverse transcribed to cDNA using a QuantiTect Reverse Transcription Kit (Qiagen). Levels of mRNA expression were semiquantified by performing real-time RT-PCR in a LightCycler System (Roche Diagnostics). The PCR mixture (20 μL) included 0.5 μM of p22^phox^ primer or 1 μM of ribosomal protein L13a (RPL13A) primer and 2× QuantiTect SYBR Green PCR Master Mix (Qiagen). PCR protocol was as follows: initial denaturation at 95° C for 5 min, followed by 40 amplification cycles of 95° C for 10 s and annealing at 60° C for 30 s. The primers used for PCR were as follows: p22^phox^ forward, 5′-CGCAGATCGGAGGCCACCATCAA-3′; p22^phox^ reverse, 5′GCGAGGTCACACGACCTCGTCG3′; HIF1A forward, 5′-TGACCAGCAACTTGAGGAAGTACCATTAT-3′; HIF1A reverse, 5′-GGTGGGTAATGGAGACATTGCCAAATTT-3′; CDH1 forward, 5′-GCCTCCTGAAAAGAGAGTGGAAG-3′; CDH1 reverse, 5′-TGGCAGTGTCTCTCCAAATCCG-3′; VIM forward, 5′-TCAGAATATGAAGGAGGAAATGGC-3′; VIM reverse, 5′-GAGTGGGTATCAACCAGAGGGA GT-3′; ZEB1 forward, 5′-GCACCTGAAGAGGACCAG AG-3′; ZEB1 reverse, 5′-GTGTAACTGCACAGGGAG CA-3′; ZEB2 forward, 5′-TTCCTGGGCTACGACCAT AC-3′; ZEB2 reverse, 5′-GCCTTGAGTGCTCGATA AGG-3′; SNAIL1 forward 5′-AGCCTGGGTGCCCTCAA GATG-3′; SNAIL1 reverse 5′-CTTGGTGCTTGTGGAG CAGGGAC-3′; RPL13A forward, 5ʹ-CCTGGAGGAGAA GAGGAAAG-3ʹ; and RPL13A reverse, 5ʹ-TTGAGGACC TCTGTGTATTT-3ʹ. mRNA levels of each gene were expressed as the ratio of RPL13A mRNA levels.

### Western blotting

Total protein was extracted using M-PER Mammalian Protein Extraction Reagent (Thermo Fisher Scientific) from cultured cells. Following measurement of protein concentration (Protein Assay Rapid Kit Wako; Wako Pure Chemical Industries, Osaka, Japan), the total protein was individually subjected to SDS-PAGE (SuperSep Ace; Wako, Japan). These proteins were transferred onto Hybond P polyvinylidene difluoride membrane (GE Healthcare, Buckinghamshire, UK). Then the proteins on the membrane were blocked in 5% nonfat dry skim milk powder (Wako) for over 1 h at room temperature and were incubated with primary antibodies for 24 h at 4° C using Immuno Shot (Cosmo Bio, Tokyo, Japan). The dilutions of primary antibodies used in this study were as follows: p22^phox^, 1:100;HIF-1α,1:200; E-Cadherin, 1:200; Vimentin, 1:200; β-actin, 1:1000. These antibody–protein complexes on the blot were detected using ECL-plus Western blotting detection reagents (GE Healthcare) following incubation with anti-rabbit or anti-mouse IgG horseradish peroxidase (GE Healthcare) at room temperature.

### RNA interference

siRNA targeting p22^phox^ used in this study was purchased from GeneDesign, Inc (Osaka, Japan). : sip22^phox^ sense, 5ʹ- UAGUAAUUCCUGGUAAAGGTT-3ʹ; sip22^phox^ anti-sense, 5ʹ- CCUUUACCAGGAAU UACUATT-3ʹ; siHIF-1α sense, 5′- GCCACUUCGA AGUAGUGCUTT-3′; and siHIF-1α antisense, 5′- AGCACUACUUCGAAGUGGCTT-3′. Silencer Select Negative Control 1 siRNA (Ambion, Austin, TX, USA) served as a negative control (siNC). siRNA 5 nM was transfected into cells (1 × 105 cells/mL) using Lipofectamine RNAi MAX reagent (Invitrogen, Carlsbad, CA, USA) for 48 or 72 h. Knockdown efficiency was assessed by RT–PCR or western blotting.

### Plasmid transfection

HIF-1α expression construct was obtained by PCR amplification of the HIF-1α coding sequence using primer sequences: forward, 5′-AAAGCTAGCATTCACCATGGAGGGCGCC-3′ and reserve, 5′-AAAGGTACCCTCAGTTAACTTGATCCAAAGC-3′. The PCR fragments were digested with KpnI and NheI, inserted into KpnI/NheI site of pcDNA3.0 (-) vector (Thermo Fisher Scientific) and then sequenced. The PCR products were cloned into KpnI/NheI site of pcDNA3.0 vector, generating pcDNA3.0-HIF-1α construct. Generation of HIF-1α stable lines PC9 cells were seeded into 10 cm culture dishes at a density of 1 × 106 cells per dish. After attachment, the cells were transfected with HIF-1α (10 μg) construct or empty vector using Lipofectamine^®^ 3000 (Thermo Fisher Scientific). At two days after transfection, the cells were re-plated in a 10 cm culture dish with medium containing 0.8 mg/ml G418. At two weeks later, the cells were sorted. The isolated colonies were seeded initially into each well of the 24-well plates, followed by progressive expansion of the cultures in large culture dishes. G418-resistant stable clones were established and maintained in medium with 0.8 mg/ml G418. Stable clones with high HIF-1α expression (PC9/pHIF-1α) were verified by qRT-PCR.

### Reactive oxygen species assay

Intracellular ROS generation was measured using DCFDA / H2DCFDA - Cellular Reactive Oxygen Species Detection Assay Kit (Abcam). These assays were carried out by plating 25,000 cells per well of each cells, respectively, into black 96-well plates. The following day, each cells was incubated with DCFDA (25 μM) for 45 minutes at 37° C in the dark. After incubation, fluorescence was measured immediately using GLOMAX MULTI+ Detection system (Promega, Madison, WI).

### Patients and tissue preparation

Surgical pathology specimens of 53 lung adenocarcinoma were retrieved from surgical patients from 2014 to 2015 at the department of Thoracic Surgery, Miyagi Cancer Center (Miyagi, Japan). The patients did neither receive chemotherapy nor radiation prior to surgery. In addition, we used six patients with lung adenocarcinoma having T790M mutation, of which four acquired EGFR-TKI resistance and underwent biopsy, and two showed primary T790M mutation in surgically resected specimens without EGFR-TKI treatment. All these six specimens were retrieved in Tohoku University Hospital (Miyagi, Japan) from 2017 to 2018. The study was conducted in accordance with the Declaration of Helsinki. The protocol was approved by the ethics committee at Tohoku University, and all patients provided informed consent.

### Immunohistochemistry

We used mouse monoclonal antibody against p22^phox^ [E-11] (Santa Cruz), Vimentin and E-cadherin. Histofine Kit (Nichirei) based on the streptavidin–biotin amplification method was used for p22^phox^ in this study. Antigen retrieval for p22^phox^ was performed using a microwave treatment with 1M citric acid buffer. The primary antibody was diluted by 1:1000. Antigen–antibody complexes were visualized using 3, 3′-diaminobenzidine solution (1 mM DAB, 50 mM Tris–HCl buffer (pH 7.6), and 0.006% H2O2) and counterstaining with hematoxylin. Human tonsil tissue was used as a positive control. Immunohistochemistry (Envision DAKO) was employed to detect the expressions of Vimentin and E-cadherin. The primary antibody was diluted by 1:200 for Vimentin and 1:500 for E-cadherin. Human normal stomach and breast tissue were used for positive control of Vimentin and E-cadherin, respectively.

Immunoreactivity of both p22^phox^ and vimentin was evaluated in cytoplasm [20], and that of E-cadherin in cell membrane. We divided the cases into three groups according to the positive rate of p22^phox^: 0% (negative), 1-50% (low expression), 50% > (high expression).

### Statistical analysis

Statistical analysis was performed using JMP Pro 14.0.0 (SAS Institute, Cary, NC, USA). Statistical differences between the two groups of immunohistochemical analyses were evaluated by *t*-test or Fisher tests. Statistical significance was defined as *p* < 0.05 in this study.

## SUPPLEMENTARY MATERIALS AND FIGURES



## Abbreviations

EGFR: epidermal growth factor receptor
LUAD: lung adenocarcinoma
EGFR–TKIs: EGFR–tyrosine kinase inhibitors
T790M: threonine-to-methionine substitution on codon 790
HIF-1α: Hypoxia Inducible Factor-1α
EMT: Epithelial-Mesenchymal Transition
PI3K: phosphoinositide 3-kinase
NADPH: nicotinamide adenine dinucleotide phosphate
NOX: NADPH oxidase
ROS: reactive oxygen species
ERK1/2: extracellular signal-regulated kinase 1/2
CDDP: cisplatin
NSCLC: non-small cell lung cancer
PEM: pemetrexed
ZEB: Zinc finger E-box-binding homeobox.
