# Supplementary material for: The correlation of p22phox and chemosensitivity in EGFR-TKI resistant lung adenocarcinoma
Source: Oncotarget. 2019 Feb 1;10(10):1119–31. doi: 10.18632/oncotarget.26637 (PMC6383684; doi:10.18632/oncotarget.26637)
Supplement: Supplementary file 1 [file oncotarget-10-1119-s001.pdf]

The correlation of p22[phox] and chemosensitivity in EGFR-TKI resistant lung adenocarcinoma

SUPPLEMENTARY MATERIALS

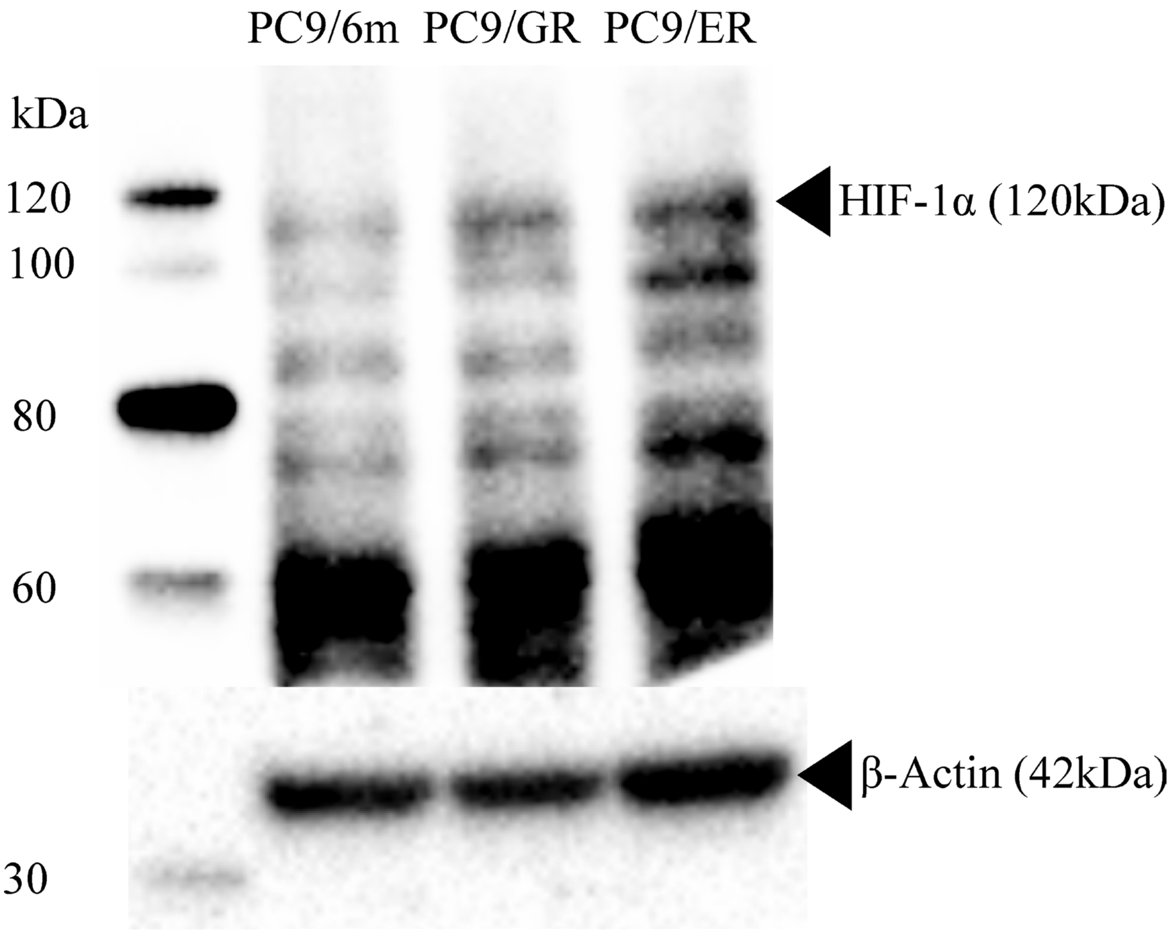

Supplementary Figure 1:HIF-1α and β-Actin western-band with the same membrane. HIF-1α and β-Actin proteins were determined using western blot analysis.

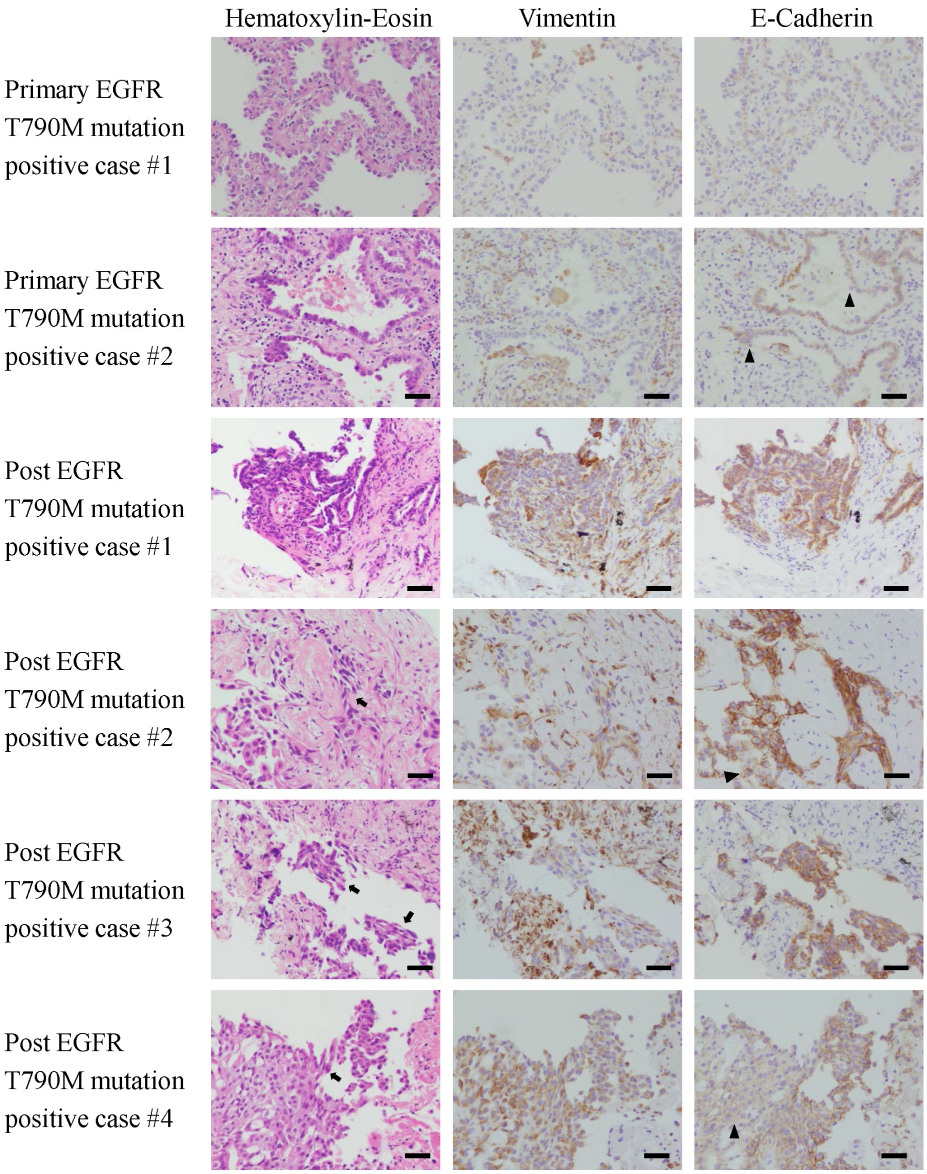

Supplementary Figure 2: Six cases which harbored T790M mutated EGFR and p22^{phox} immunopositivity. These cases were stained by Hematoxylin-Eosin and immunohistochemistry against Vimentin and E-cadherin were performed. Two cases with primary T790M mutated EGFR (#1, 2) demonstrated weakened immunoreactivity for E-cadherin (arrow head), although they did not show apparent spindle-shaped tumor cells and immunoreactivity for vimentin. All four cases with acquired T790M mutated EGFR demonstrated immunoreactivity for vimentin, three of them showed focally spindle-shaped tumor cells (#2, 3, 4) (arrow), and two of them showed weakened immunoreactivity for E-cadherin (#2, 4) (arrow head). Bar: 50 μm.
